# Supplementary material for: CemR atypical response regulator impacts energy conversion in Campylobacteria
Source: mSystems. 2024 Jul 9;9(8):e00784-24. doi: 10.1128/msystems.00784-24 (PMC11334517; doi:10.1128/msystems.00784-24)
Supplement: Supplemental material — Figures S1 to S13, Tables S1 and S2, and descriptions of Data S1 to S3. [file msystems.00784-24-s0004.pdf]

Supplemental Material

CemR atypical response regulator impacts energy conversion in *Campylobacteria*

This PDF file includes:

Supplementary Tables

Tab. S1: Strains, plasmids and proteins used in this study.

Tab. S2: Primers used in this study.

Supplementary Figures

Fig. S1: Disc diffusion assay of *C. jejuni* and *A. butzleri* indicating the influence of Cj1608 and Abu0127 on resistance to oxidative agents.

Fig. S2: RNA-seq analysis of *C. jejuni* gene transcription controlled by Cj1608.

Fig. S3: RNA-seq analysis of *A. buzleri* gene transcription controlled by Abu0127.

Fig. S4: LC-MS/MS analysis of *C. jejuni* and *A. butzleri* protein level regulation mediated by Cj1608 and Abu0127, respectively.

Fig. S5: Pearson correlation analysis between proteomics and transcriptomics data of *C. jejuni* and *A. butzleri*.

Fig. S6: The KEGG pathway enrichment of *C. jejuni* citrate cycle.

Fig. S7: The KEGG pathway enrichment of *A. butzleri* oxidative phosphorylation and citrate cycle.

Fig. S8: Transcriptomics and proteomics data of citrate synthetase operon.

Fig. S9: Transcriptomics and proteomics data of NADH-quinone oxidoreductase operon.

Fig. S10: Impact of HP1021 on gene regulation in *H. pylori*.

Fig. S11: *C. jejuni* NCTC 11168 ΔCj1608 knock-out mutant construction.

Fig. S12: *A. butzleri* RM4018 ΔAbu0127 knock-out mutant construction

Fig. S13: The reproducibility of *C. jejuni* and *A. butzleri* biological replicates in omics data.

Description of Additional Supplemental Materials

Supplemental Material References

Table S1: Strains, plasmids and proteins used in this study.

Strain	Relevant features	Reference/source
E. coli DH5α	*supE44*, *hsdR17*, *recA1*, *endA1*, *gyrA1*, *gyrA96*, *thi-1*, *relA1*	(1)
E. coli BL21	F-, *ompT*, *hsdS (rB-, mB-)*, *gal*, *dcm*	GE Healthcare
C. jejuni NCTC 11168	Parental strain	(2)
C. jejuni NCTC 11168 ΔCj1608	Δ*Cj1608*::*aphA-3*; 11168 with *Cj1608* exchanged to *aphA-3* cassette	This study
C. jejuni NCTC 11168 C_{Cj1608}	(Δ*Cj1608*::*aphA-3*)::*Cj1608*, *hdsM*::*cat*; 11168 ΔCj1608 in which *aphA-3* was exchanged to *Cj1608* and *cat* cassette was inserted in to *hsdM* gene	This study
A. butzleri RM4018	Parental strain	(3), DSMZ-German Collection of Microorganisms and Cell Cultures GmbH
A. butzleri RM4018 ΔAbu0127	Δ*Abu0127*::*aphA-3*; Abu0127 exchanged to *aphA-3* cassette	This study

Plasmid	Relevant features	Reference/source
pUC18	Cloning vector, Amp^R	Thermo Fisher Scientific
pCR2.1-TOPO®	TA cloning vector, Amp^R, Kan^R	Thermo Fisher Scientific
pCR2.1/ΔCj1608	pUC18 derivative containing *Cj1608* flanking regions and *aphA-3* for allelic exchange of *Cj1608* for *aphA-3*	This study
pTZ57R/TΔHP1021	pTZ57R/T derivative containing *aphA-3* gene	(4)
pUC18/COM/Cj1608	pUC18 derivative containing *Cj1608* flanking regions and *Cj1608* for allelic exchange of *aphA-3* for *Cj1608*	This study
pSB3021	Suicide vector for integration of *cat* gene in *hsdM* gene for complementation	(5)
pUC18/ΔAbu0127	pUC18 derivative containing *Abu0127* flanking regions and *aphA-3* for allelic exchange of *Abu0127* for *aphA-3*	(6)
pET28/StrepCj1608	pET28Strep derivative containing the *Cj1608* gene for protein expression	This study
pET28/StrepAbu0127	pET28Strep derivative containing the *Abu0127* gene for protein expression	This study

Recombinant protein	Relevant features	Reference/source
StrepCj1608	Recombinant, *C. jejuni* Cj1608 protein, Strep-tagged at N-terminus, purified from *E. coli*	This study
StrepAbu0127	Recombinant, *A. butzleri* Abu0127 protein, Strep-tagged at N-terminus, purified from *E. coli*	This study

Table S2: Primers used in this study.

Oligo name	Sequence (5' → 3')
**P1**	gatcaaaacctagaatttacagatg
**P2**	cacccgggtaccgagtcataatcttgtccaatcaaaatatt
**P3**	tggacaagattatgactcggtacccgggtgactaa
**P4**	ctgatttcatatttcttcctttctaaaacaattcatccagtaaaatatag
**P5**	ctatattttactggatgaattgttttagaaaggaagaaatatgaaatcag
**P6**	ctgctttatcaatagtccaacg
**P7**	cagatacaacactttttgacaatg
**P8**	gctcgacatactgttcttccc
**P9**	acagctatatccagcatcacttag
**P10**	gtcgactctagaggatccccggatttaaagcacatagtgatgg
**P11**	ctcctagttagtcaggatcctcatcttttttccaattataatat
**P12**	ggatcctgactaactaggaggaataaatg
**P13**	ctaaaacaattcatccagtaaaat
**P14**	ttttactggatgaattgttttaggggataatgtatgaatttgagaaa
**P15**	cgaattcgagctcggtacccccctgcaataatatcatcaccc
**P16**	ttttgacattcatgcctttgaagag
**P17**	gataaaccgcagctgttgcaatt
**P18**	gtttgttttcagcaagccactc
**P19**	gccaagcgttgtagatatgtca
**P23**	aaaccaccagaacaggcaca
**P24**	tgacgtttcaagatttggagcag
**P25**	taccataacccatagcaccgat
**P26**	agaaattgaaggcgatatgggc
**P27**	tctggaccttgaacaaattgca
**P28**	agccttcggttcttcctaca
**P29**	cgggatccatgaaagttttaattattgaaaatg
**P30**	ccggtcgacttatttttttcttgctgatttcata
**P31**	cgggatccatgaacatattaattatcgaaaatg
**P32**	ccggtcgacctatttcttttttcgatatctag
**P33**	ggagtaagaatagcttcgaatggcccagttgtaccgtcatata
**P34**	ctttattcagcgcttattttaacagc
**P35**	ggagtaagaatagcttcgaatgaatagctgctcttgaagcagc
**P36**	cgctatcaaataccatgcgac
**P37**	ggagtaagaatagcttcgaatcaatgaaaattgtggaagct
**P38**	agattgaatacagctttgagt
**P39**	ggagtaagaatagcttcgaattccacttgttataagttcatcattt
**P40**	acttgatattagaagaattgcaact
**P41**	FAM-ggagtaagaatagcttcgaat*
**P42**	Cy5- ggagtaagaatagcttcgaat**
**P43**	cggaatttgacattttcgctcc
**P44**	gcggttatttgaggctattattgttatga
**P45**	gtggaagctaatattaattccgttgaca
**P46**	tgtttcttttggtagcaacgga

* - FAM, 6-fluorescein amidite;

** - Cy5, tetramethylindo(di)-carbocyanines 5.

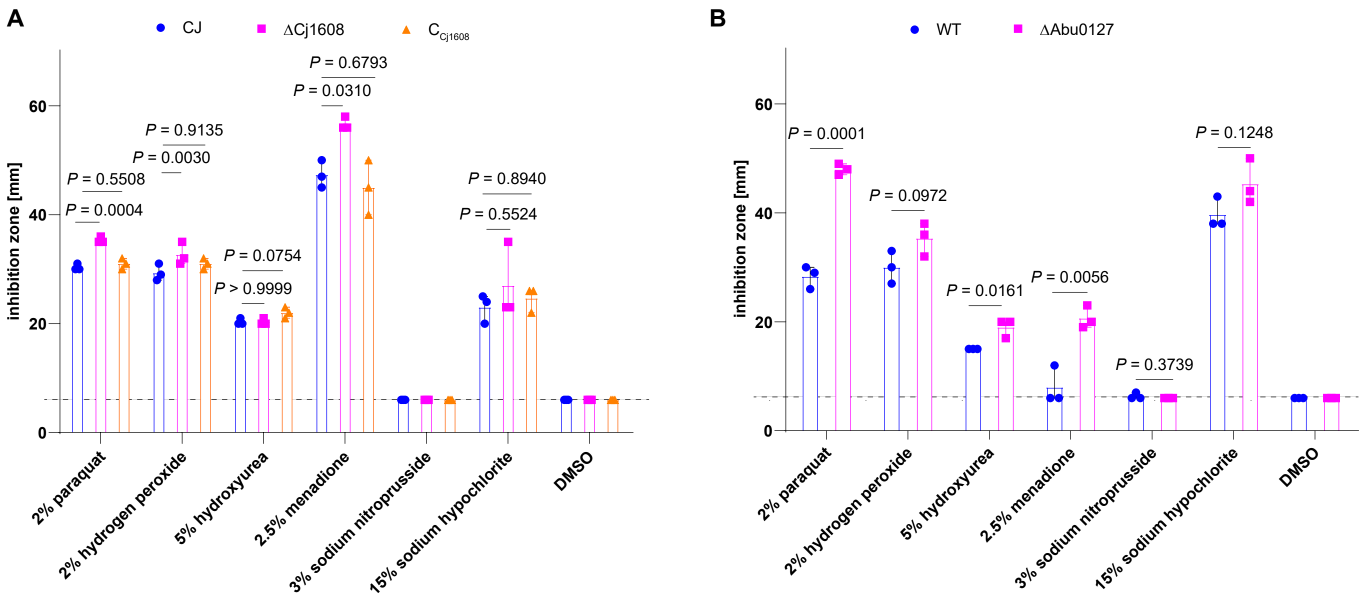

S1 Fig. Disc diffusion assay of *C. jejuni* and *A. butzleri* indicating the influence of Cj1608 and Abu0127 on resistance to oxidative agents. (A) *C. jejuni* wild-type (CJ)), Cj1608 knock-out mutant (ΔCj1608) and Cj1608 complementation (C_{Cj1608}), and **(B)** *A. butzleri* wild-type (AB) and Abu0127 knock-out mutant (ΔAbu0127) strains were analyzed. The black dashed lines indicate the disc size. Data have been depicted as the mean values ± SD. n = 3 biologically independent experiments. **(A)** Ordinary one-way ANOVA with Tukey's multiple comparison test determined the P value. **(B)** The students' paired t-test determined the P value. Data taken from (6).

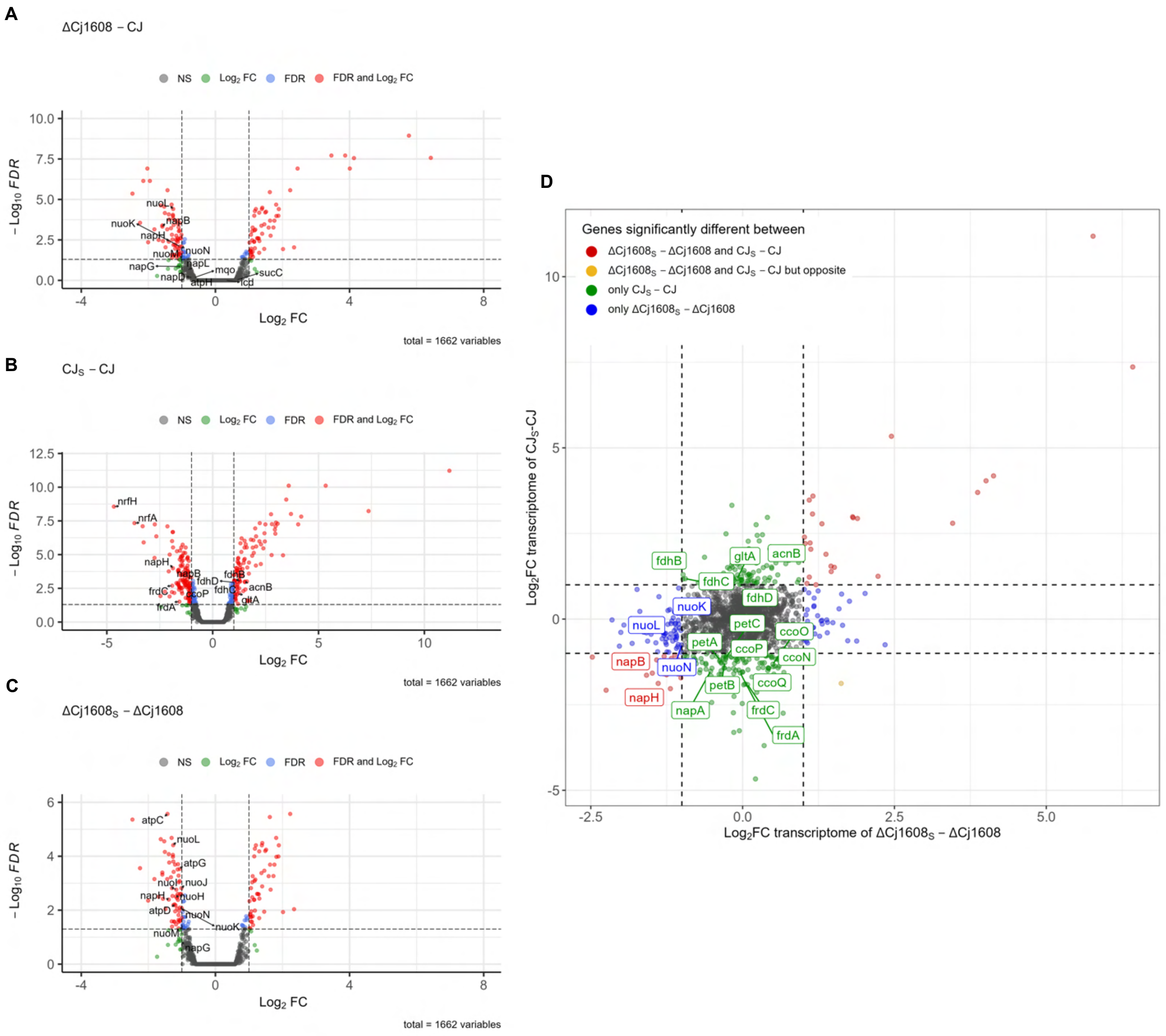

S2 Fig. RNA-seq analysis of *C. jejuni* gene transcription controlled by Cj1608. (A) The Volcano plot of genes differently transcribed in the Cj1608 knock-out mutant (ΔCj1608) strain compared to the *C. jejuni* wild-type (CJ) strain (ΔCj1608-CJ). **(B)** The Volcano plot of genes differently transcribed in the CJ strain under oxidative stress induced by 1 mM paraquat (CJ_S) compared to the non-stressed wild-type strain (CJ_S-CJ). **(C)** Volcano plot of genes differently transcribed in the ΔCj1608 strain under oxidative stress induced by 1 mM paraquat ($\Delta Cj1608_S$) compared to the non-stressed ΔCj1608 mutant ($\Delta Cj1608_S$-ΔCj1608). **(D)** The comparison of gene transcription in the *C. jejuni* CJ, CJ_S, ΔCj1608 and stressed $\Delta Cj1608_S$ cells revealed by RNA-seq. The genes signed on the graph correspond to the citric acid cycle and electron transport chain. **(A-D)** Values outside the black dashed lines indicate a change in the expression of $|\log_2 FC| \geq 1$. Grey dots correspond to genes whose transcription was not significantly changed (FDR ≤ 0.05). Genes whose transcription significantly changed ($|\log_2 FC| \geq 1$; FDR ≤ 0.05) are depicted by colored dots; see the legend in the figure.

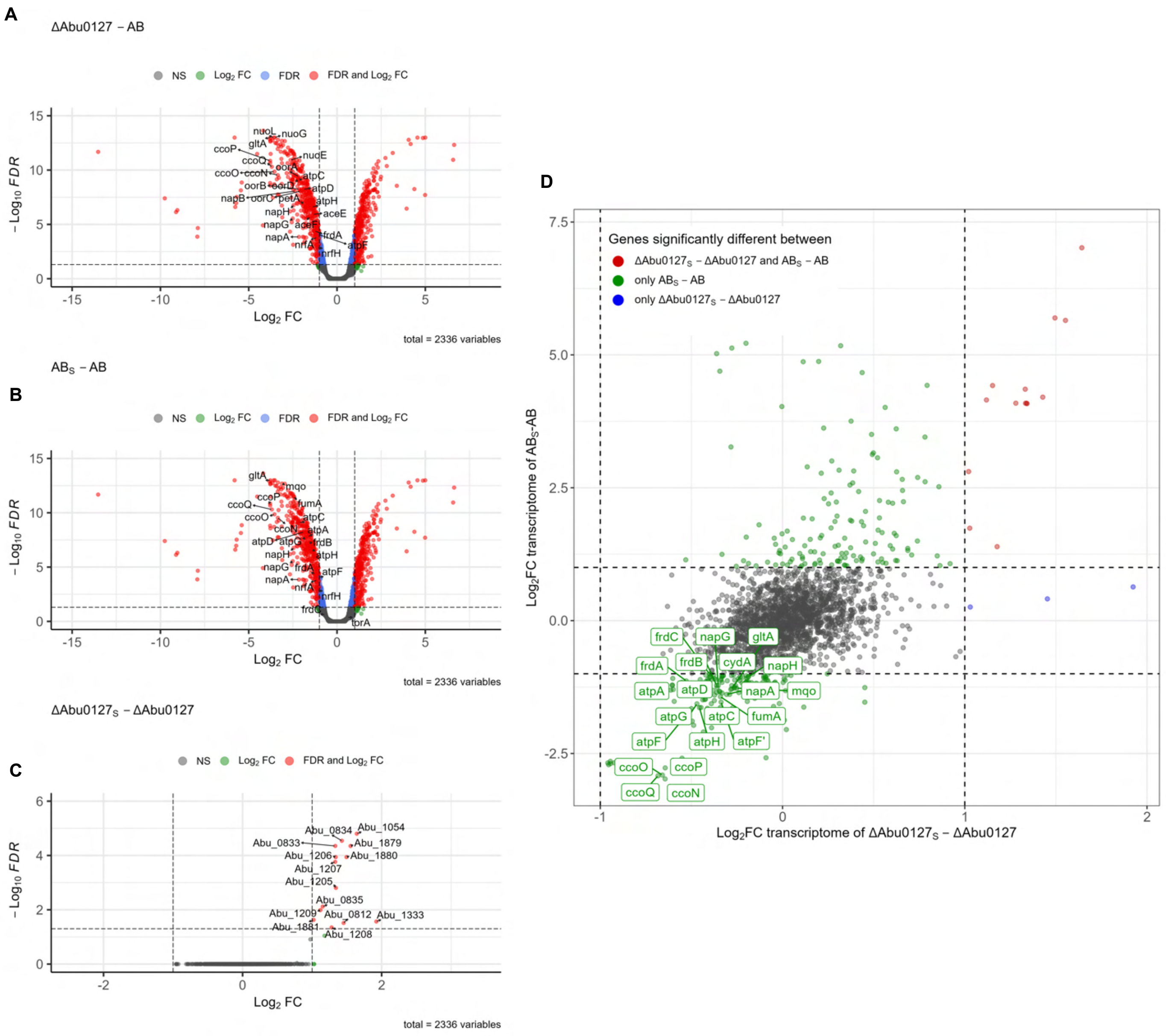

S3 Fig. RNA-seq analysis of *A. butzleri* gene transcription controlled by Abu0127. (A) The Volcano plot of genes differently transcribed in the Abu0127 knock-out mutant (ΔAbu0127) strain compared to the *A. butzleri* wild-type (AB) strain(ΔAbu0127-AB). **(B)** The Volcano plot of genes differently transcribed in the AB strain under oxidative stress induced by 1 mM paraquat (AB_S) compared to the non-stressed wild-type strain (AB_S-AB). **(C)** Volcano plot of genes differently transcribed in the ΔAbu0127 strain under oxidative stress induced by 1 mM paraquat ($ΔAbu0127_S$) compared to the non-stressed ΔAbu0127 mutant ($ΔAbu0127_S$-ΔAbu0127). **(D)** The comparison of gene transcription in the *A. butzleri* AB, AB_S, ΔAbu0127 and stressed $ΔAbu0127_S$ cells revealed by RNA-seq. The genes signed on the graph correspond to the citric acid cycle and electron transport chain. **(A-D)** Values outside the black dashed lines indicate a change in the expression of $|log_2FC| \geq 1$. Grey dots correspond to genes whose transcription was not significantly changed (FDR ≤ 0.05). Genes whose transcription significantly changed ($|log_2FC| \geq 1$; FDR ≤ 0.05) are depicted by colored dots; see the legend in the figure.

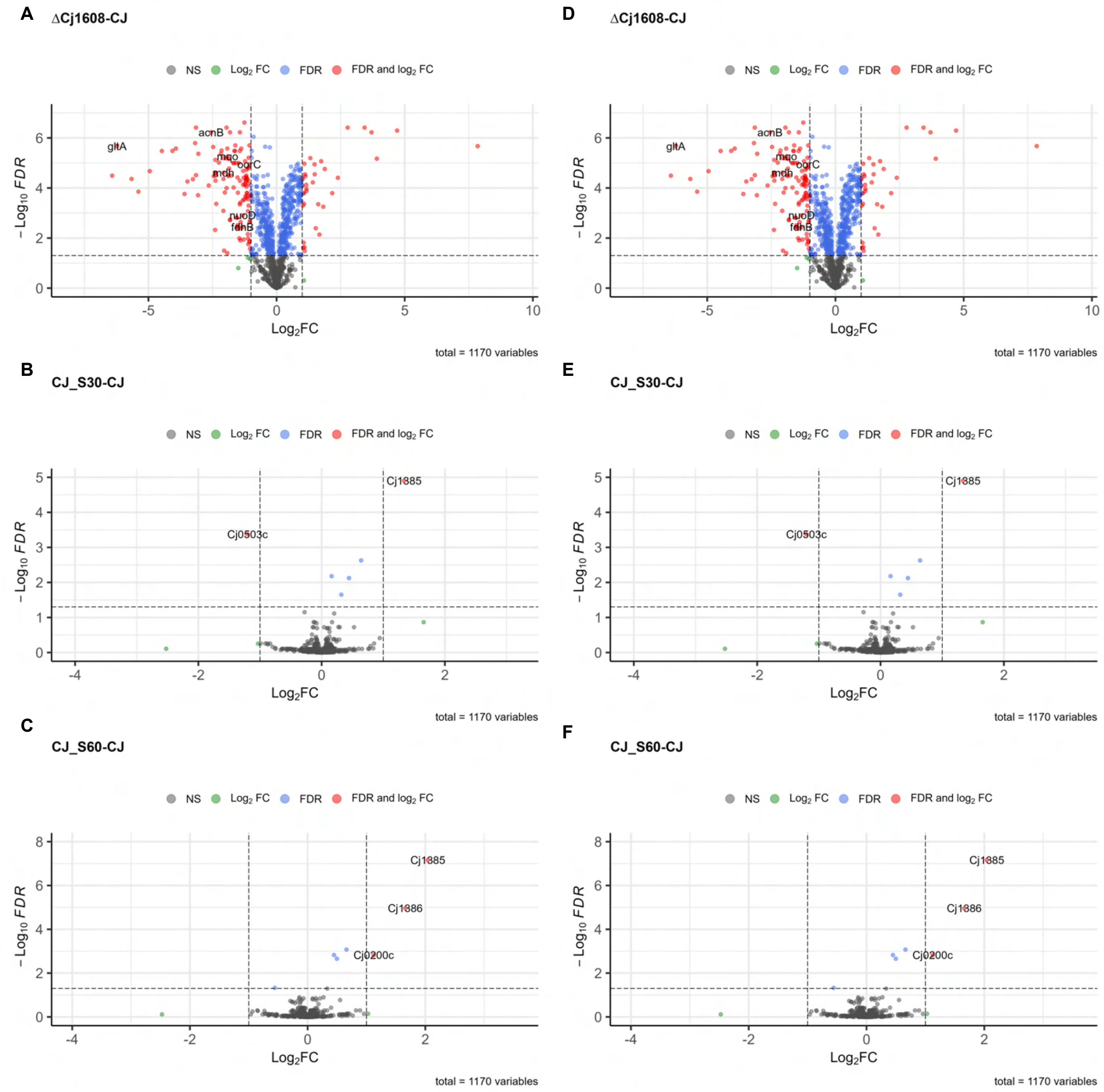

S4 Fig. LC-MS/MS analysis of *C. jejuni* and *A. butzleri* protein level regulation mediated by Cj1608 and Abu0127, respectively. (A) Volcano plot of proteins differently expressed in the Cj1608 knock-out mutant (ΔCj1608) strain compared to the *C. jejuni* wild-type (CJ) strain (ΔCj1608-CJ). **(B)** Volcano plot of proteins differentially expressed in the CJ strain after 30-min oxidative stress (CJ_S30) induced by 1 mM paraquat compared to the non-stressed wild-type strain (CJ_S30-CJ). **(C)** Volcano plot of proteins differentially expressed in the CJ strain after 60 min of oxidative stress (CJ_S60) induced by 1 mM paraquat compared to the non-stressed CJ strain (CJ_S60-CJ). **(D)** Volcano plot of proteins differently expressed in the Abu0127 knock-out mutant (ΔAbu0127)strain compared to the *A. butzleri* wild-type (AB) strain (ΔAbu0127-AB). **(E)** Volcano plot of proteins differentially expressed in the AB strain after 30-min oxidative stress (AB_S30) induced by 1 mM paraquat compared to the non-stressed AB strain (AB_S30-AB). **(F)** Volcano plot of proteins differentially expressed in the AB strain after 60 min of oxidative stress (AB_S60) induced by 1 mM paraquat compared to the non-stressed AB strain (AB_S60-AB). **(A-F)** n = 4 biologically independent experiments. Green dots correspond to genes with $|log_2FC| \geq 1$ and FDR ≥ 0.05; blue dots correspond to genes with $|log_2FC| \leq 1$ and FDR ≤ 0.05; red dots correspond to genes with $|log_2FC| \geq 1$ and FDR ≤ 0.05; grey dots correspond to genes that were not significantly changed. NS, non-significant. FDR, false discovery rate.

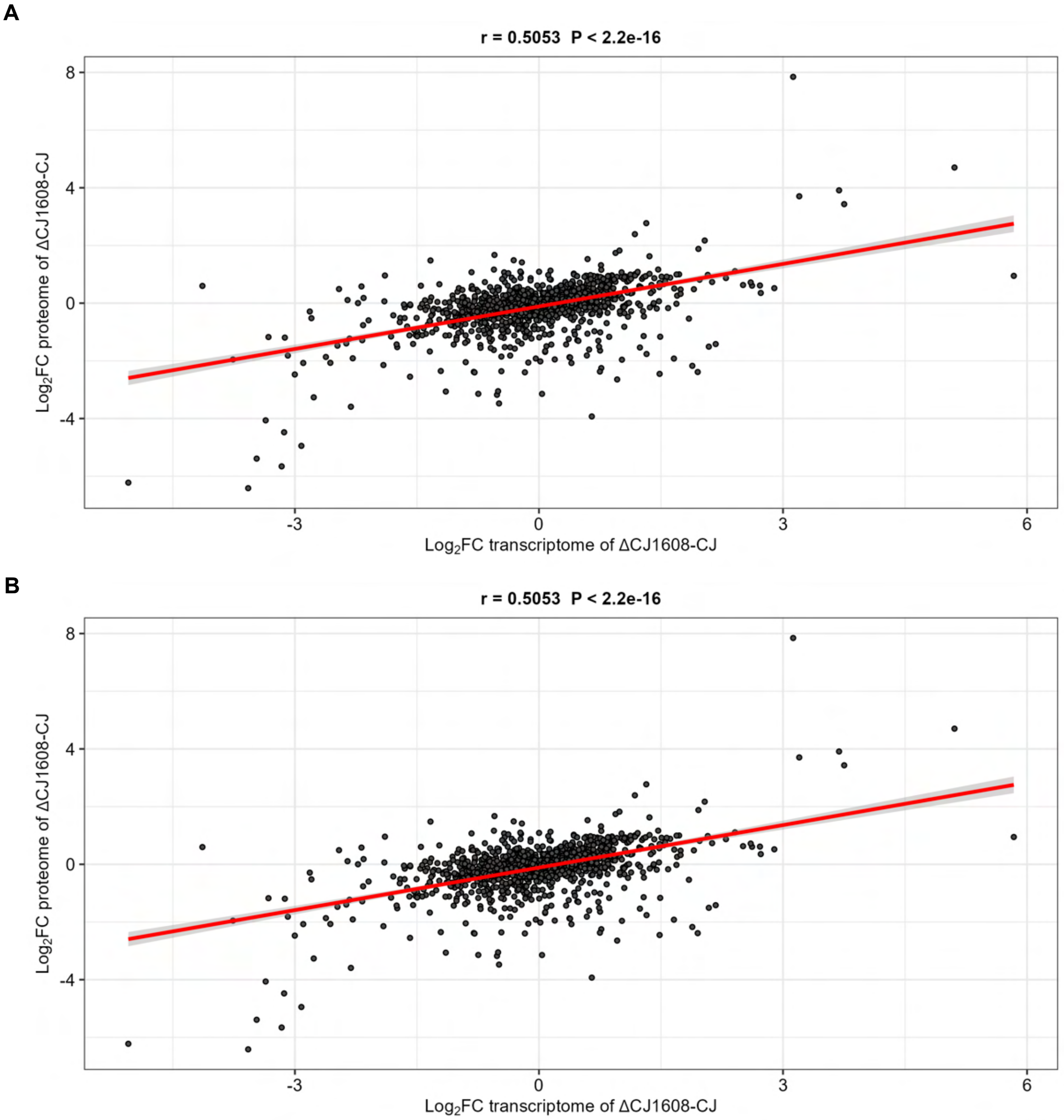

S5 Fig. Pearson correlation analysis between proteomics and transcriptomics data of (A) *C. jejuni* and **(B)** *A. butzleri*. Scatter plots of the correlation between data sets of the log_2FC of gene transcript (x-axis) and proteome (y-axis) of ΔCj1608-CJ and ΔAbu0127-AB, respectively. The red line represents the regression line; r, Pearson correlation coefficient.

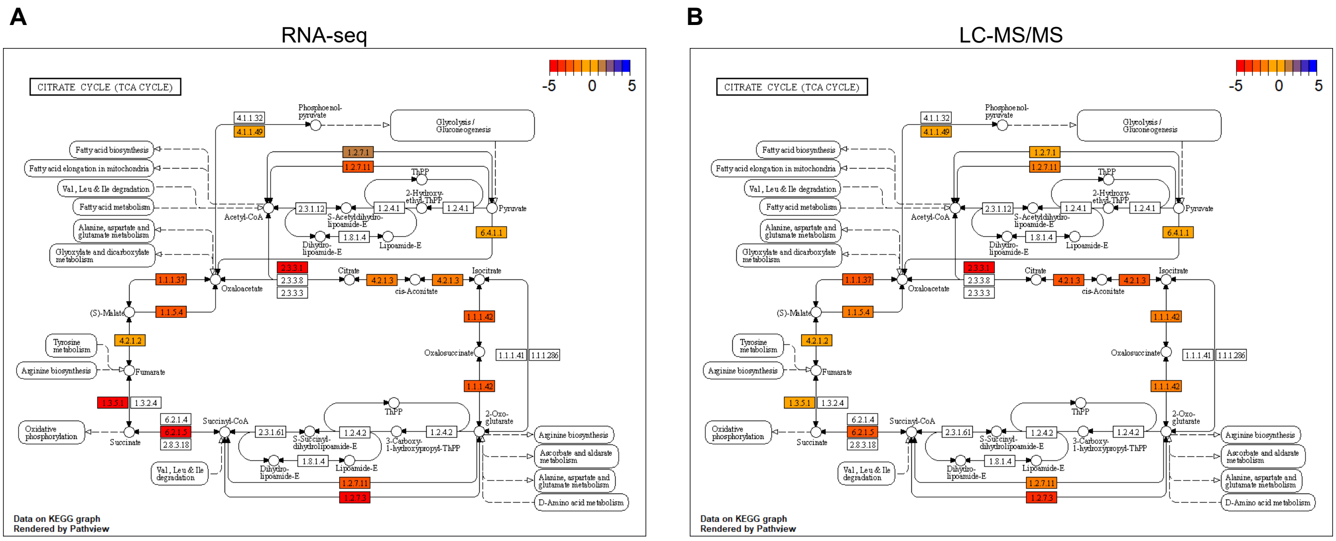

S6 Fig. The KEGG pathway enrichment of *C. jejuni* citrate cycle. (A) Gene enrichment of the citrate cycle KEGG group visualized by ClusterProfiler. Gene expression values are mapped to a gradient color scale of $\log_2$FC. **(B)** Protein enrichment of the citrate cycle KEGG group visualised by ClusterProfiler. Protein expression values are mapped to a gradient color scale of $\log_2$FC.

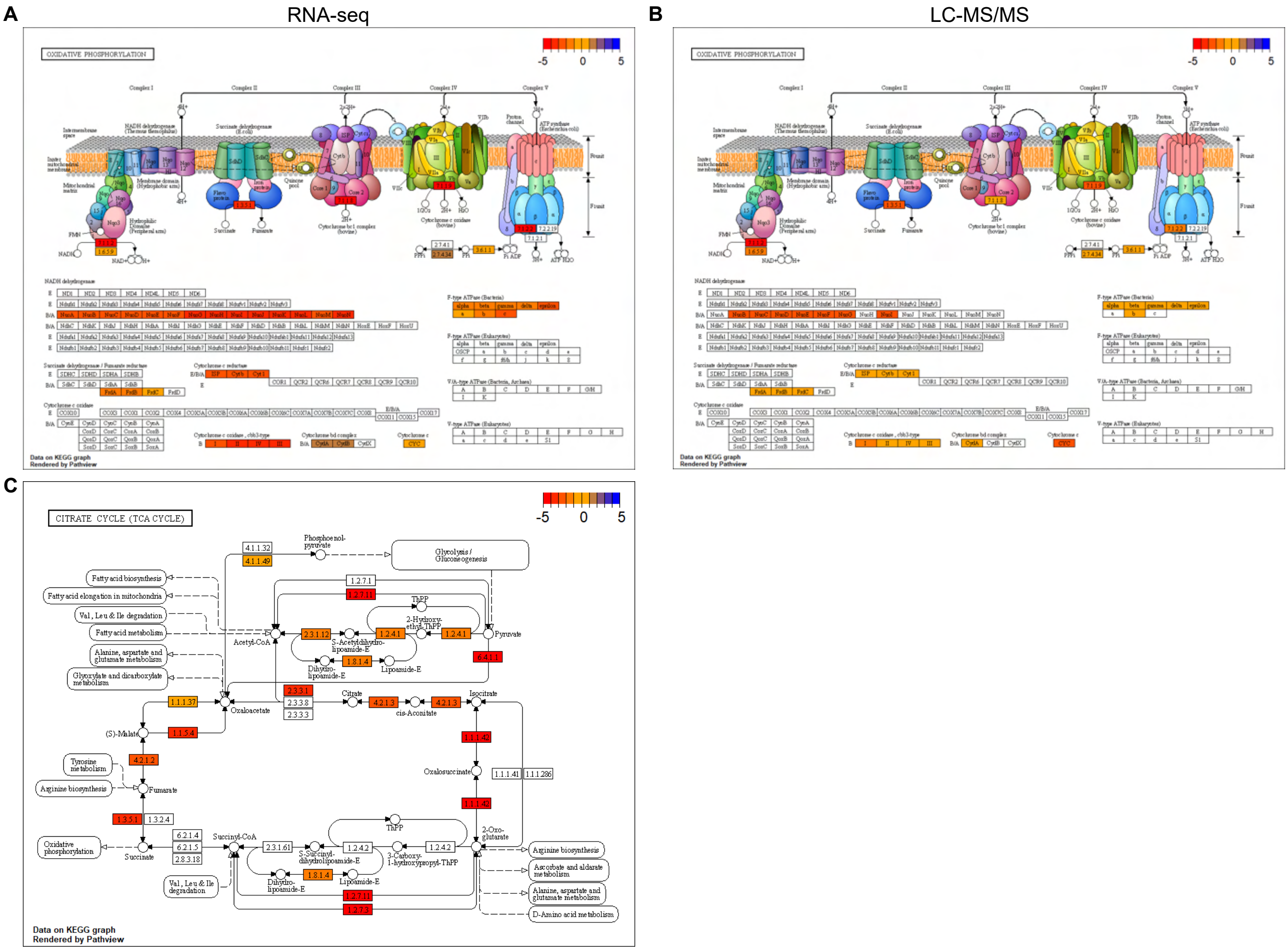

S7 Fig. The KEGG pathway enrichment of *A. butzleri* oxidative phosphorylation and citrate cycle. (A) Gene enrichment of oxidative phosphorylation KEGG group visualized by ClusterProfiler. Gene expression values are mapped to a gradient colour scale of $\log_2$FC. **(B)** Protein enrichment of oxidative phosphorylation KEGG group visualized by ClusterProfiler. Protein expression values are mapped to a gradient colour scale of $\log_2$FC. **(C)** Gene enrichment of the citrate cycle KEGG group visualized by ClusterProfiler. Protein expression values are mapped to a gradient color scale of $\log_2$FC.

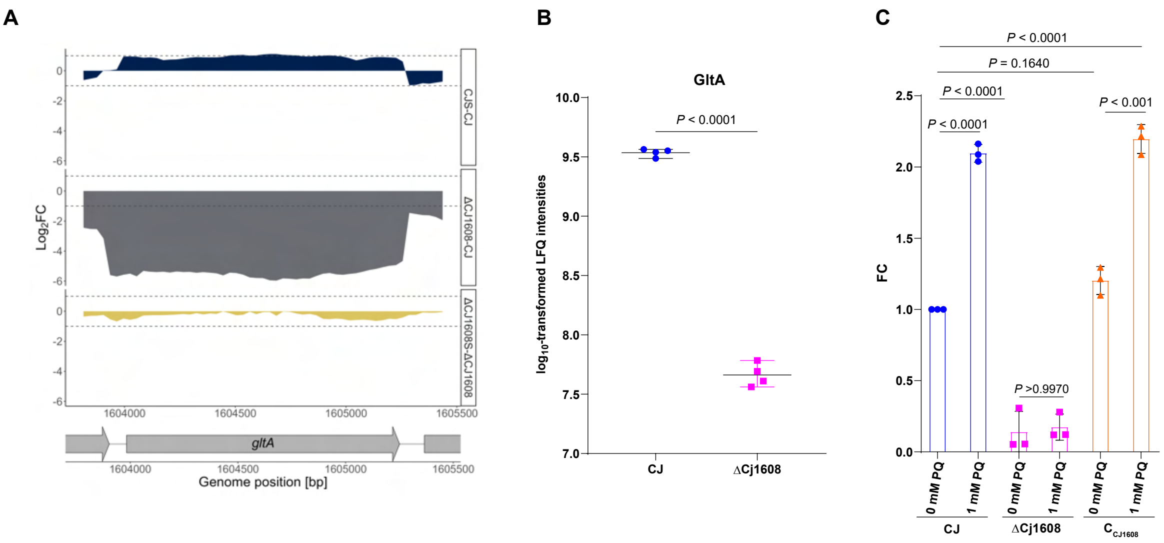

S8 Fig. Transcriptomics and proteomics data of citrate synthetase operon. (A) RNA-seq data profile comparing *gltA* transcription in *C. jejuni* wild-type (CJ), Cj1608 knock-out mutant (ΔCj1608) strains under microaerobic growth and paraquat-induced oxidative stress. Values above the black dashed lines indicate a change in the expression of $|\log_2 FC| \geq 1$; FDR ≤ 0.05. **(B)** Logarithmically transformed LFQ intensities of GltA protein in CJ and ΔCj1608 strains. The student's unpaired t-test determined the P value. n = 4 biologically independent experiments. **(C)** RT-qPCR analysis of *gltA* transcription in CJ, ΔCj1608 and Cj1608 complementation (C_{Cj1608}) strains cells cultured under microaerobic or paraquat-induced oxidative stress conditions. n = 3 biologically independent experiments. Data presented as the mean values ± SD. Ordinary one-way ANOVA with Tukey's multiple comparison test determined the P value.

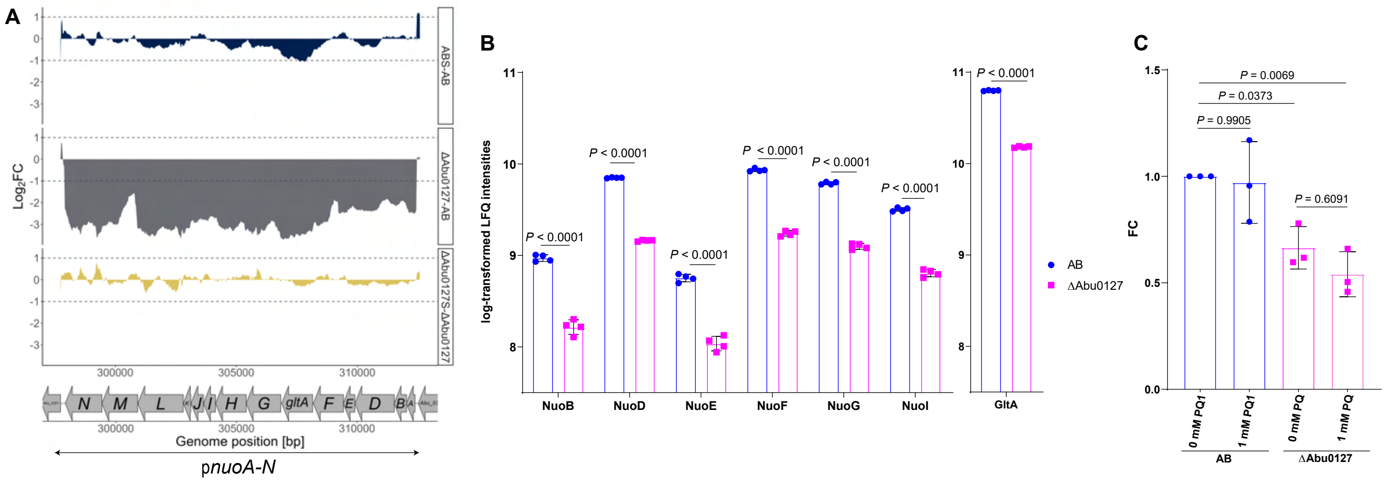

S9 Fig. Transcriptomics and proteomics data of NADH-quinone oxidoreductase operon. (A) RNA-seq data profile comparing the transcription of the *nuo* operon in *A. butzleri* wild-type (AB) and Abu0127 knock-out mutant (ΔAbu0127) strains under microaerobic growth and paraquat-induced oxidative stress Values above the black dashed lines indicate a change in the expression of $|\log_2 FC| \geq 1$; FDR ≤ 0.05. **(B)** Logarithmically transformed LFQ intensities of Nuo B-F, GltA, NuoG, and NuoI proteins in AB and ΔAbu0127 strains. n = 4 biologically independent experiments. **(C)** RT-qPCR analysis of *nuoB* transcription in AB and ΔAbu0127 cells cultured under microaerobic or paraquat-induced oxidative stress conditions. **(B and C)** Data presented as the mean values ± SD. Ordinary one-way ANOVA with Tukey's multiple comparison test determined the P value. n = 3 biologically independent experiments.

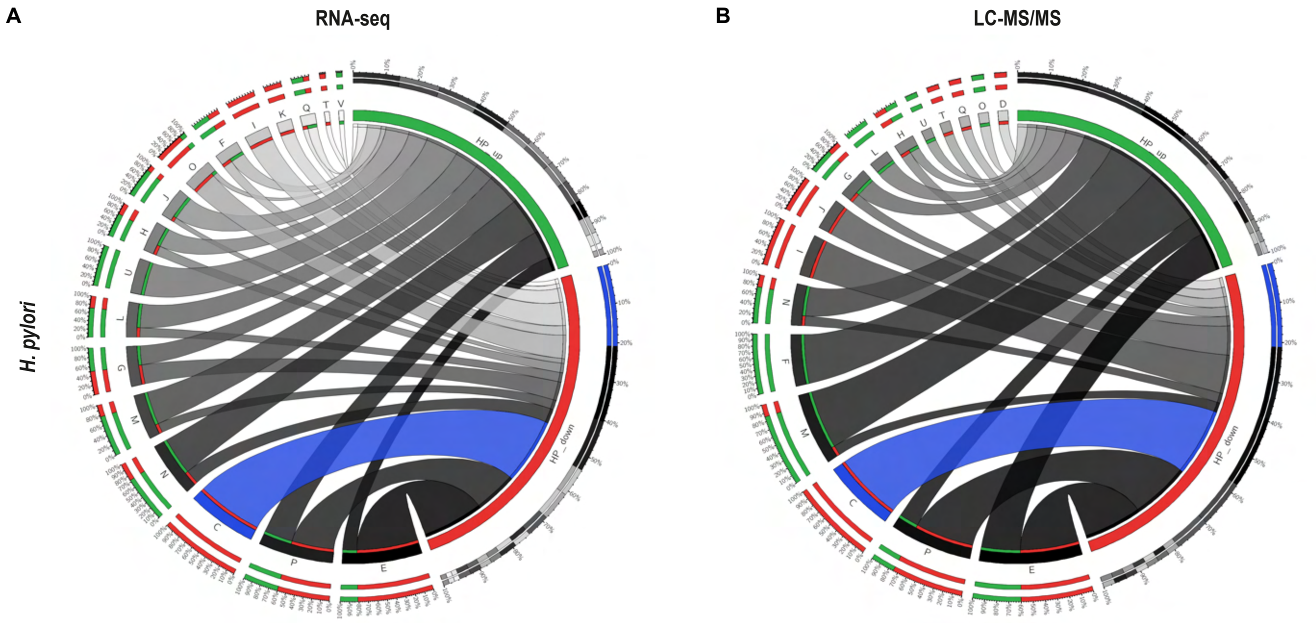

C - energy production and conversion, **D** - cell cycle control, cell division, chromosome partitioning, **E** - amino acid transport and metabolism, **F** - nucleotide transport and metabolism, **G** - carbohydrate transport and metabolism, **H** - coenzyme transport and metabolism, **I** - lipid transport and metabolism, **J** - translation, ribosomal structure and biogenesis, **K** - transcription, **L** - replication, recombination and repair, **M** - cell wall/membrane/envelope biogenesis, **N** - cell motility, **O** - posttranslational modification, protein turnover, chaperones , **P** - inorganic ion transport and metabolism, **Q** - secondary metabolites biosynthesis, transport and catabolism, **T** - signal transduction mechanisms, **U** - intracellular trafficking, secretion, and vesicular transport, **V** - defense mechanisms

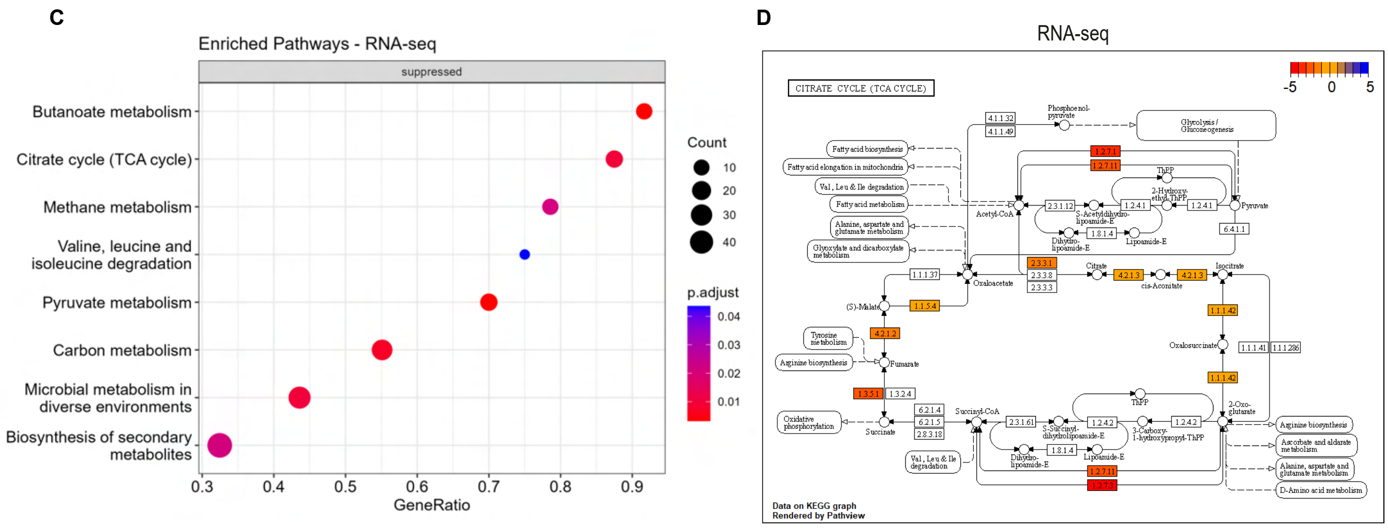

S10 Fig. Impact of HP1021 on gene regulation in *H. pylori*. (A) Circos plots presenting the correlation between Cluster of Orthologues Group (COG) and differentially expressed *H. pylori* genes revealed by RNA-seq (|$\log_2$FC| ≥ 1 and FDR ≥ 0.05) in the *HP1021* knock-out mutant (ΔHP1021) strain compared to the *H. pylori* wild-type strain. **(B)** Circos plots presenting the correlation between COG and differentially expressed *H. pylori* proteins revealed by LC-MS/MS (|$\log_2$FC| ≥ 1 and FDR ≥ 0.05) in the ΔHP1021 strain compared to the *H. pylori* wild-type strain. **(C)** ClusterProfiler gene enrichment plot showing suppression of KEGG groups revealed by RNA-seq in the ΔHP1021 strain compared to the *H. pylori* wild-type strain. **(D)** Gene enrichment of citrate cycle KEGG group visualized by ClusterProfiler. Gene expression values are mapped to a gradient color scale of $\log_2$FC. **(A-D)** Data prepared based on (7).

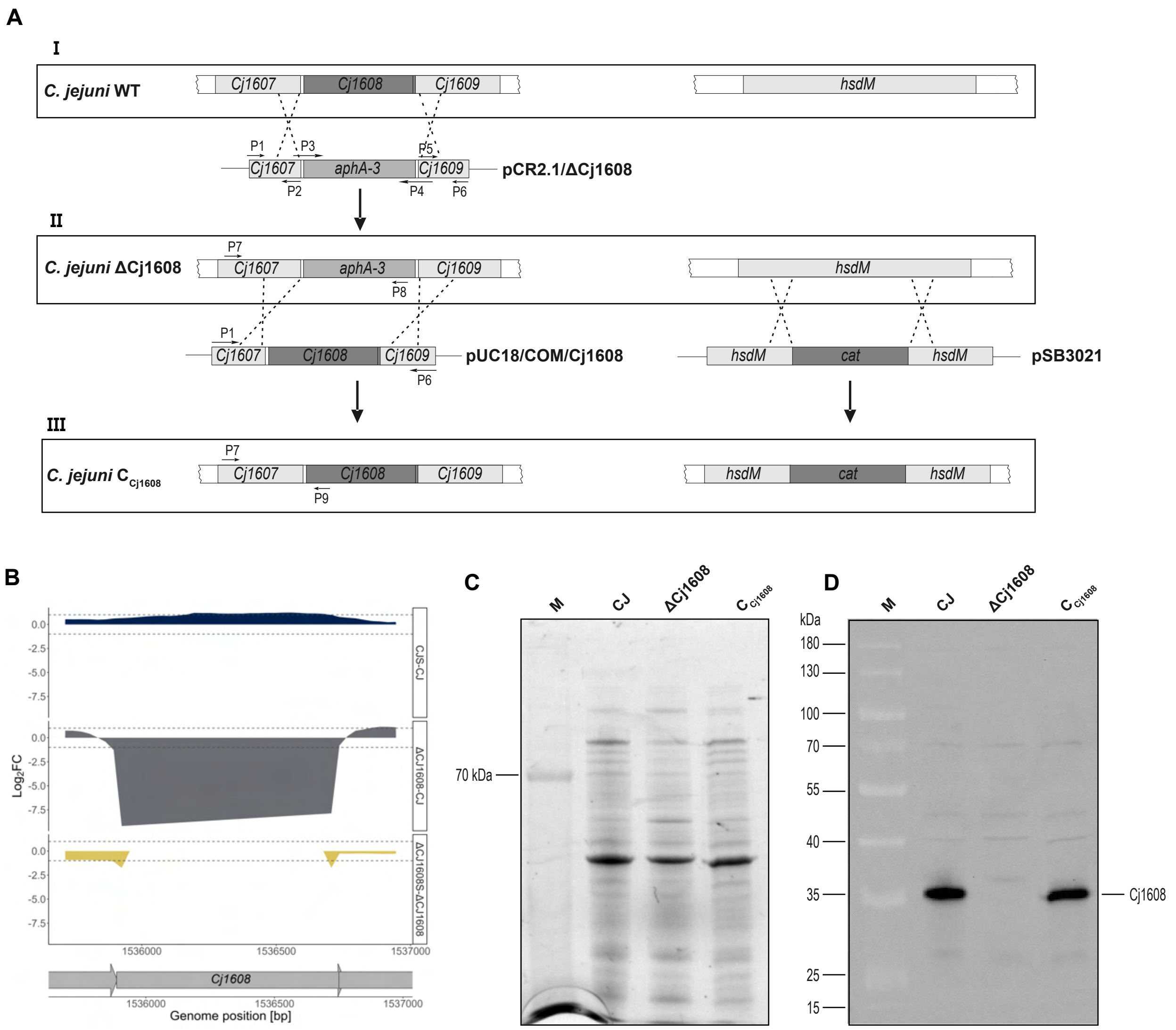

S11 Fig. *C. jejuni* NCTC 11168 ΔCj1608 knock-out mutant construction. (A) The mutagenesis strategy used to delete and subsequently complement Cj1608 on the *C. jejuni* chromosome. For the plasmids and primer sequences, see S1 Table and S2 Table, respectively. **(B)** RNA-seq data profile of Cj1608 gene of *C. jejuni* wild-type (CJ) and ΔCj1608 strains with the expression comparison. **(C)** Western blot analysis of Cj1608 in CJ and ΔCj1608 and complementation (C_{Cj1608}) strains. Lysate of each strain was resolved in a 10% SDS-PAGE gel and visualized by the TCE-UV method. **(D)** Cj1608 was detected in bacterial lysates by a rabbit polyclonal anti-StrepCj1608 antibody. M, PageRuler Prestained Protein Ladder (Thermo Fisher Scientific). Digital processing was applied equally across the entire image.

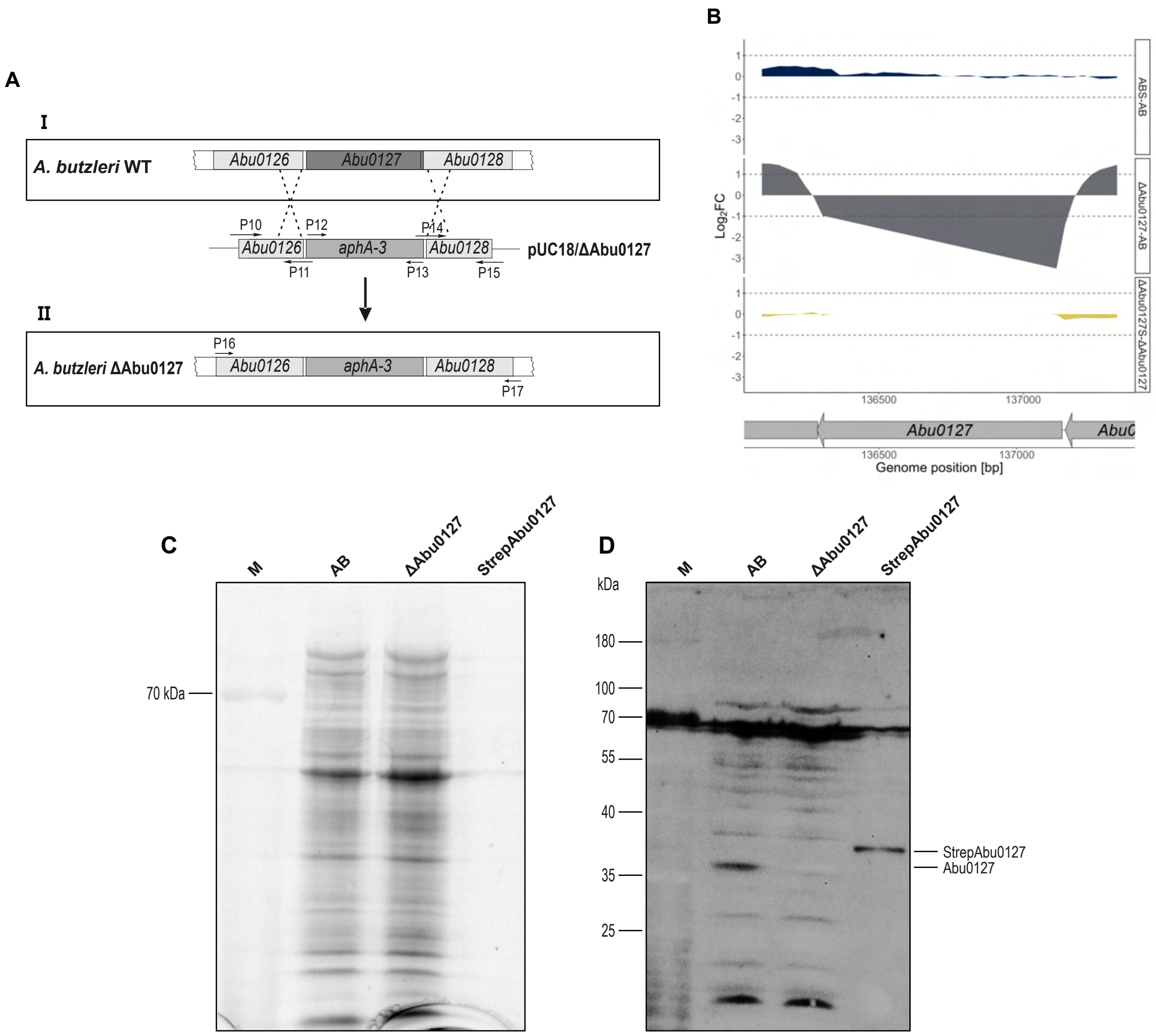

S12 Fig. *A. butzleri* RM4018 ΔAbu0127 knock-out mutant construction. (A) The mutagenesis strategy used to delete Abu0127 on the *A. butzleri* chromosome. For the plasmids and primer sequences, see S1 Table and S2 Table, respectively. **(B)** RNA-seq data profile of Abu0127 gene in *A. butzleri* wild-type (AB) and ΔAbu0127 strains with the expression comparison. **(C)** Western blot analysis of Abu0127 in AB and ΔAbu0127 strains. Lysate of each strain was resolved in a 12% SDS-PAGE gel and visualized by the TCE-UV method. 2.5 ng of StrepAbu0127 was used as a positive control. **(D)** Abu0127 and StrepAbu0127 were detected by a rabbit polyclonal anti-StrepAbu0127 antibody. M, PageRuler Prestained Protein Ladder (Thermo Fisher Scientific). Digital processing was applied equally across the entire image.

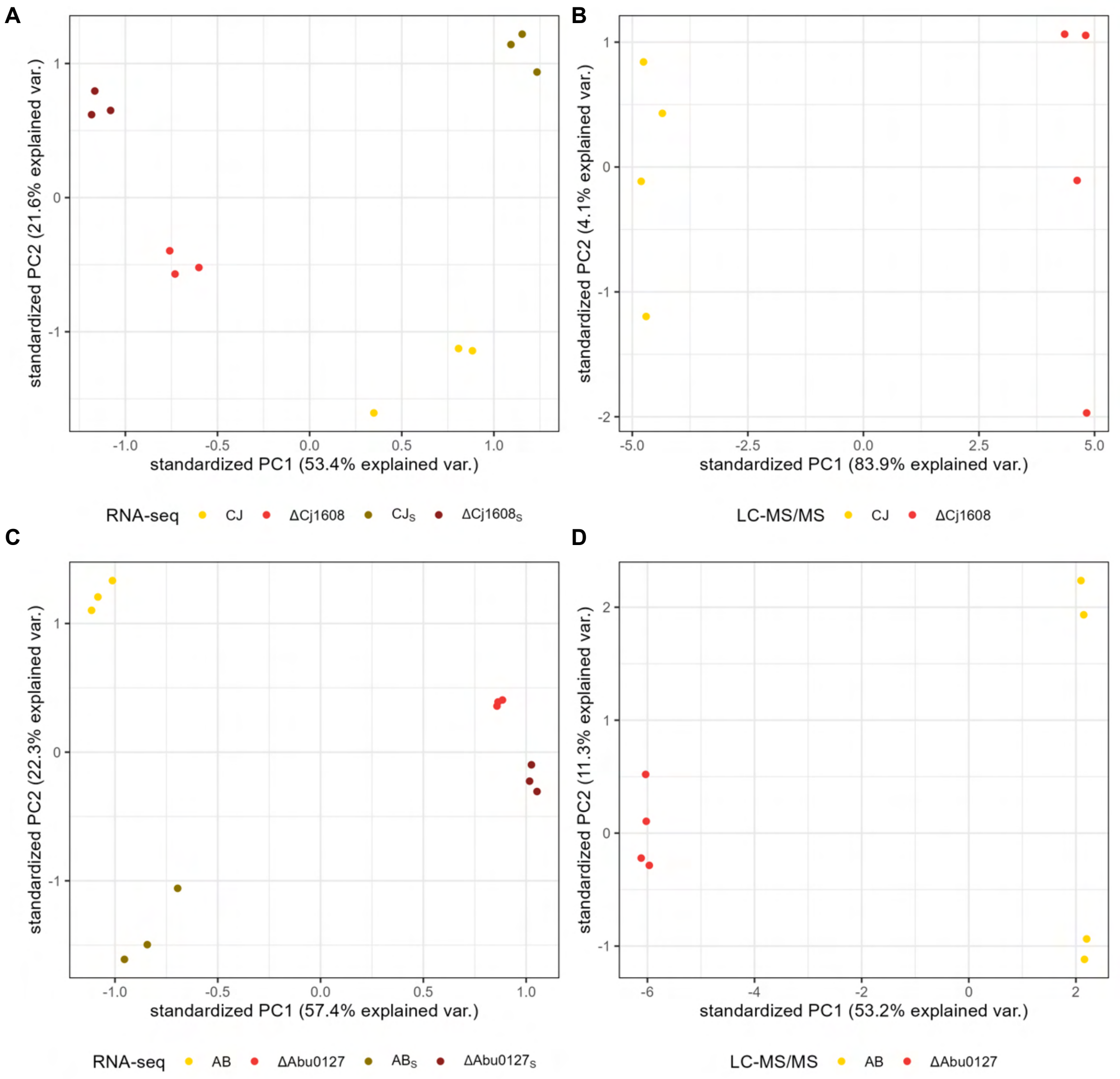

S13 Fig. The reproducibility of *C. jejuni* and *A. butzleri* biological replicates in omics data. (A) Principal component analysis (PCA) of the normalized RNA-seq CPM data of *C. jejuni* wild-type (CJ) and Cj1608 knock-out mutant (ΔCj1608) strains under microaerobic and paraquat-induced oxidative stress conditions (CJ_S and $\Delta Cj1608_S$). **(B)** PCA of the normalized LC-MS/MS CPM data of CJ and ΔCj1608 strains under microaerobic conditions. **(C)** PCA of the normalized RNA-seq CPM data of *A. butzleri* wild-type (AB) and Abu0127 knock-out mutant (ΔAbu1027) strains under microaerobic and paraquat-induced oxidative stress conditions (AB_S and $\Delta Abu0127_S$). **(D)** PCA of the normalized proteomics CPM data of AB and ΔAbu1027 strains under microaerobic conditions.

Description of Additional Supplemental Materials

S1 Data. Analysis of Cj1608-dependent gene expression in *C. jejuni* WT and Cj1608 knock-out mutant (ΔCj1608) strains under microaerobic and oxidative stress conditions. RNA-seq and LC-MS/MS data are presented. Genes are annotated according to the *C. jejuni* NCTC 11168 strain (NC_002163.1).

S2 Data. Analysis of Abu0127-dependent gene expression in *A. butzleri* WT and Abu0127 knock-out mutant (ΔAbu0127) strains under microaerobic and oxidative stress conditions. RNA-seq and LC-MS/MS data are presented. Genes are annotated according to the *A. butzleri* RM4018 strain (NC_009850.1).

S3 Data. Comprehensive RNA-seq and LC-MS/MS data results for genes of selected processes or pathways in *C. jejuni* and *A. butzleri*. Part of the data was compared to *H. pylori* omics data from (7). Transcriptomic and proteomic data are presented as $\log_2$-fold changes. Legends specific to each pathway/process were added below particular tables.

Supplemental Material References

1. Sambrook J, Russel DW. 2001. Molecular Cloning: A Laboratory ManualCold Spring Harbor Laboratory Press. Cold Spring Harbor Laboratory Press, New York.
2. Parkhill J, Wren BW, Mungall K, Ketley JM, Churcher C, Basham D, Chillingworth T, Davies RM, Feltwell T, Holroyd S, Jagels K, Karlyshev A V., Moule S, Pallen MJ, Penn CW, Quail MA, Rajandream MA, Rutherford KM, Van Vliet AHM, Whitehead S, Barrell BG. 2000. The genome sequence of the food-borne pathogen Campylobacter jejuni reveals hypervariable sequences. Nature 2000 403:6770 403:665–668.
3. Miller WG, Parker CT, Rubenfield M, Mendz GL, Wösten MMSM, Ussery DW, Stolz JF, Binnewies TT, Hallin PF, Wang G, Malek JA, Rogosin A, Stanker LH, Mandrell RE. 2007. The Complete Genome Sequence and Analysis of the Epsilonproteobacterium Arcobacter butzleri. PLoS ONE 2.
4. Szczepanowski P, Noszka M, Zdot;yła-Uklejewicz D, Pikuła F, Nowaczyk-Cieszewska M, Krężel A, Stingl K, Zawilak-Pawlik A. 2021. HP1021 is a redox switch protein identified in Helicobacter pylori. Nucleic Acids Research 49:6863–6879.
5. Watson RO, Novik V, Hofreuter D, Lara-Tejero M, Galán JE. 2007. A MyD88-Deficient Mouse Model Reveals a Role for Nramp1 in Campylobacter jejuni Infection. Infection and Immunity 75:1994.
6. Cieślak M. 2021. Construction of the plasmid for the analysis of the essentiality of the Abu0127 gene for the survival of Arcobacter butzleri RM4018. Wroclaw University of Science and Technology .
7. Noszka M, Strzałka A, Muraszko J, Kolenda R, Meng C, Ludwig C, Stingl K, Zawilak-Pawlik A. 2023. Profiling of the Helicobacter pylori redox switch HP1021 regulon using a multi-omics approach. Nature Communications 2023 14:1 14:1–14.
